# Cancer Vaccine by Fusions of Dendritic and Cancer Cells

**DOI:** 10.1155/2009/657369

**Published:** 2010-02-18

**Authors:** Shigeo Koido, Eiichi Hara, Sadamu Homma, Yoshihisa Namiki, Toshifumi Ohkusa, Jianlin Gong, Hisao Tajiri

**Affiliations:** ^1^Division of Gastroenterology and Hepatology, Department of Internal Medicine, The Jikei University School of Medicine, Tokyo 277-8567, Japan; ^2^Institute of Clinical Medicine and Research, The Jikei University School of Medicine, Tokyo 277-8567, Japan; ^3^Department of Oncology, Institute of DNA Medicine, The Jikei University School of Medicine, Tokyo 277-8567, Japan; ^4^Saitama Cancer Center Research Institute for Clinical Oncology, Saitama 277-8567, Japan; ^5^Department of Medicine, Boston University School of Medicine, Boston, MA 02118, USA

## Abstract

Dendritic cells (DCs) are potent antigen-presenting cells and play a central role in the initiation and regulation of primary immune responses. Therefore, their use for the active immunotherapy against cancers has been studied with considerable interest. The fusion of DCs with whole tumor cells represents in many ways an ideal approach to deliver, process, and subsequently present a broad array of tumor-associated antigens, including those yet to be unidentified, in the context of DCs-derived costimulatory molecules. DCs/tumor fusion vaccine stimulates potent antitumor immunity in the animal tumor models. In the human studies, T cells stimulated by DC/tumor fusion cells are effective in lysis of tumor cells that are used as the fusion partner. In the clinical trials, clinical and immunological responses were observed in patients with advanced stage of malignant tumors after being vaccinated with DC/tumor fusion cells, although the antitumor effect is not as vigorous as in the animal tumor models. This review summarizes recent advances in concepts and techniques that are providing new impulses to DCs/tumor fusions-based cancer vaccination.

## 1. Cancer Vaccine

Cancer vaccine is treatment that enhances the patient's own immune system. The antigen-presenting cells (APCs) most suitable for cancer vaccine are dendritic cells (DCs), which can be distinguished from B cells and macrophages by their abundant expression of costimulatory molecules and ability to initiate a strong primary immune response [1]. A major area of investigation in cancer vaccine involves the design of DC-based cancer vaccines [2]. DCs are specialized to capture and process tumor-associated antigens (TAAs), converting the proteins to peptides that are presented on major histocompatibility complex (MHC) class I and class II molecules. DCs then migrate to T-cell areas of secondary lymphoid organs and become competent to present antigens to T cells, thus initiating antigen-specific immune responses [1, 2]. Ex vivo generated, antigen-loaded DCs have been used as vaccines to improve immunity. But there is considerable controversy as to which forms of antigen loading are most effective. Different strategies have been developed to load DCs with TAAs, including synthetic peptides derived from the known antigens [3], tumor lysates [4], tumor RNA [5], and dying tumor cells [6] to induce antigen-specific immune responses. These DCs have been concomitantly treated with conditioning factors such as a standard mixture of cytokines (TNF-a, IL-1*β*, IL-6, and PGE2) [7] or Toll-like receptors (TLRs) [8] that induce DC maturation, thus converting them into potent APCs. The immunogenicity of antigens delivered by DCs has been shown in patients with cancer [4]. Although clinical trials have demonstrated immunological responses after vaccination with DCs loaded with tumor specific peptides, the efficacy of therapeutic vaccination against cancer has recently been questioned because of the undeniably limited rate of objective tumor regressions that has been observed in clinical trials [9]. However, several aspects of DC vaccination require optimization to improve clinical responses including the facilitation of innate and adaptive interactions and reduction of regulatory T cell (Treg) networks or suppressive tumor-microenvironments that inhibit the function of antitumor immune responses [10].

## 2. Antitumor Immunity by DCs/Tumor Fusions in Animal Model

**Figure 1:**
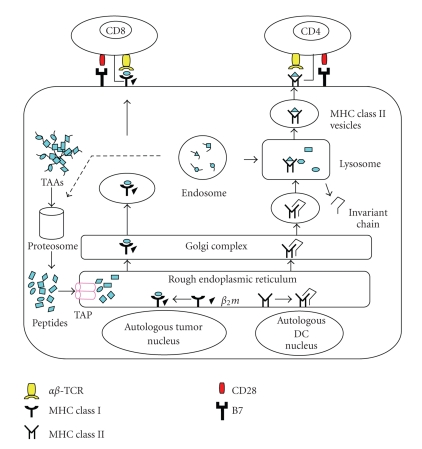
Fusions of autologous DCs and autologous tumor cells. The DCs/tumor fusion cells express MHC class I and class II and costimulatory molecules as well as tumor-associated antigens. The fusions are able to process tumor-derived peptides and MHC class I peptides derived from DCs. They form MHC class I-peptide complexes, in the endoplasmic reticulum, which are transported to the surface and presented to CD8+ T cells. Similarly, the fusion cells can also synthesize MHC class II peptides derived from DCs in the endoplasmic reticulum, which are transported to the cytoplasm where MHC class II-peptide complexes are assembled with tumor-derived peptides. These complexes are presented to CD4+ T cells, which are important for efficient CTL induction.

Effective delivery of antigens into DCs is an important aspect for clinical trials. Vaccination with DCs loaded with tumor specific peptides has been used [3, 4, 11]. However, a major drawback of this strategy comes from a limited number of known tumor peptides available in many HLA contexts and the potential evasion of immunological targeting through downregulation of their antigens. To solve this problem, an alternative approach has been developed by fusing DCs with tumor cells [12]. In this approach, a broad spectrum of TAAs, including those known and unidentified, can be fully presented on MHC class I and class II molecules in the context of costimulatory molecules (Figure 1) [13, 14]. 

The fusion of syngeneic DCs and tumor cells creates a heterokaryon with both tumor-derived antigens and DCs-derived MHC class II costimulatory molecules (B7.1 and B7.2), intracellular adhesion molecule- (ICAM-) 1, lymphocyte function-associated antigen- (LFA-) 1 and -3, and CD40, all of which are efficient antigen-processing and presentation machinery [15, 16]. Ex vivo generated DCs can be fused with whole tumor cells and reinfused to the patients [17], or they can be used for ex vivo induction and expansion of cytotoxic T lymphocytes (CTLs) [18, 19]. Indeed, DCs/tumor fusion cell vaccines have been shown to possess the elements essential for processing and presenting tumor antigens to host immune cells for inducing effective antitumor immune response and for breaking T-cell tolerance to tumor-associated antigens in animal models [12, 20]. Many animal studies have demonstrated that the DCs/tumor fusion vaccine not only provided protection against challenge with tumor cells, but also regressed established tumors, including melanoma [21–27], colorectal [12–14, 18, 28–35], breast [36–39], esophageal [40], pancreatic [41], hepatocellular [42–46], lung [47, 48], laryngeal [49], renal cell carcinoma [50], sarcoma [51–53], myeloma [54–59], mastocytoma [60], and neuroblastoma [61].

In our initial study on DCs/tumor fusion cell vaccines, murine MC38 adenocarcinoma cells stably transfected with human MUC1 (MC38/MUC1) were fused to synergistic DCs derived from bone marrow in the presence of polyethylene glycol (PEG). MUC1 that is a high-molecular-weight glycoprotein that is overexpressed in breast, ovarian, and pancreatic adenocarcinomas [62] represents a potential target for active specific immunotherapy against certain human tumors [5]. We have used MUC1-transgenic (MUC1. Tg) mice that express MUC1 as a self-protein on normal ductal epithelial cells. Because MUC1.Tg mice that express MUC1 in a pattern and at a level similar to that found in humans are unresponsive to MUC1 antigen, these mice provide a potential model to assess the induction of anti-MUC1 immune responses [63]. Vaccination of wild-type mice with MUC1 RNA-transfected DCs (DCs/MUC1 RNA) induced anti-MUC1 immune responses against MUC1-positive MC38/MUC1, but not MUC1-negative tumor cells. In contrast, there is little if any anti-MUC1 immunity induced with the DCs/MUC1 RNA in MUC1.Tg mice [5]. Interestingly, vaccination with fusions of DCs and MC38/MUC1 tumor cells induced effective cellular and humoral immunity against MUC1 antigen in MUC1.Tg mice. The fusion vaccine provided protection against challenge with MUC1-positive tumor cells and mediated regression of established tumors in the mice. These findings indicate that vaccination with the fusion cells can induce CTLs against MUC1 and thereby reverse tolerance to human MUC1 antigen [20]. Therefore, DCs/tumor fusion-based vaccine may represent an effective strategy to induce antitumor immunity, including MUC1-positive tumor cells.

Although the transplantable tumor models have been contributed as the primary screening tools for cancer vaccine development, they do not fit this criterion since the tumor in these models grows very quickly, without the multiple stages of cancer development found in human cancers. On the other hand, genetically modified mice with spontaneous development of carcinoma provide a powerful tool to study the efficacy of tumor vaccines, since they mimic cancer development in humans. We have used a double transgenic mouse model expressing polyomavirus middle T oncogene and human MUC1 as self-antigen to determine the preventive effect of a DCs/tumor fusion cell vaccine. Even in the genetically altered model of spontaneous breast cancer, vaccination with DCs/tumor fusion cells conferred sufficient antitumor immunity to block or delay mammary tumor development [36, 37]. 

## 3. Antigen Presentation and Processing by DCs/Tumor Fusions

Immature DCs display a characteristic phenotype with high expression levels of MHC class I, class II, costimulatory molecules (CD80 and CD86), low levels of the maturation marker, CD83, but not TAAs. As compared with immature DCs, mature DCs expressed much higher levels of HLA-DR, CD80, CD86, and CD83. By contrast, almost all of tumors expressed an abundance of TAAs, MHC class I, but not MHC class II and costimulatory molecules. Fusion of DCs and tumor cells resulted in the formation of a heterokaryon that combined DC-derived MHC class I, class II, and costimulatory molecules, efficient antigen-processing and presentation machinery, and an abundance of tumor-derived MHC class I and antigens [12]. After fusion, the cytoplasm of DCs and tumor cells was integrated into one entry, whereas their nuclei remained separate entities [64]. Such a structure may make it possible to maintain the function of both original live cells (DCs and tumor cells), at least in part, including synthesis of TAAs, MHC class I, class II, and costimulatory molecules. Moreover, the DCs/tumor fusions also delivered not only proteins but also mRNA encoding the whole TAAs from tumor cells. The DCs/tumor fusions approach facilitates the entry of TAAs that are synthesized de novo in the fusions into the endogenous antigen-processing pathway of the DCs. Thus, the TAAs can be processed and presented through both MHC class I and class II pathways on the DCs in the context of costimulatory molecules [12]. The advantage of DCs/tumor fusion vaccine over pulsing DCs with tumor lysates is that endogenously synthesized antigens have better access to MHC class I pathway [65]. In animal studies, fusion vaccine was superior to DCs loaded with antigenic protein or peptide, tumor cell lysates, or irradiated tumor cells [25]. In human DCs/tumor fusions, it has been also demonstrated that the tumor antigens were processed through the endogenous pathway of DCs after fusion and T cells primed by the fusions were high quality antigen-specific cells, capable of mediating lysis of tumor targets [66]. In our reports, we have created hybrid cells by fusing autologous DCs and allogeneic tumor cell lines that did not express same MHC class I molecules as autologous DCs [67, 68]. These fusions expressed both MHC class I- and class II-restricted tumor-associated epitopes through the cross-priming.

## 4. Activation of Antigen-Specific CD4+ and CD8+ T Cells by DCs/Tumor Fusions

DCs reside at the port of entry, take up exogenous antigens, and migrate to draining lymph nodes, where the antigens are presented to CD4+ T cells through MHC class II pathways. In addition, DCs are capable of initiating CD8+ T cell response through a cross-presentation pathway [69, 70]. For cancer vaccination, the goal is to generate antigen-loaded DCs that efficiently stimulate robust and long-lasting CD4+ and CD8+ T cell responses in the patient with cancer, with the emphasis on “long-lasting” [71]. Importantly, vaccination with the fusion cells is associated with activation of antigen-specific CD4+ and CD8+ T cells [14, 19, 64]. To dissect the role of MHC class I- or class II-restricted antigen-specific T cell activation by the fusions, we have created various types of DCs/tumor fusion cells by alternating fusion cell partners using three kinds of knockout mice and wild type mice: (1) wild type fusions (WT-FCs), (2) MHC class I knockout fusions (IKO-FCs), (3) MHC class II knockout fusions (IIKO-FCs), and (4) MHC class I and class II double-knockout fusions (I/IIKO-FCs) [72]. In this study, immunization of MUC1.Tg mice with WT-FCs, IKO-FCs, IIKO-FCs, or I/IIKO-FCs provided 100%, 76.6%, 61.5%, and 15.4% protection, respectively, against tumor challenge with MC38/MUC1 tumor cells. This study has demonstrated that MHC class II antigen presentation targeting activation of CD4+ T cells was indispensable in antitumor immunity. The lower antitumor immunity by IIKO-FCs may be due to lack of help from MHC class II-restricted CD4+ T cells in the priming phase, whereas the induction of antitumor immunity by IKO-FCs is through cross-priming by the host DCs. The results suggest a novel mechanism of antitumor immunity mediated by CD4+ T cells [72]. Previously, much research has been devoted to the significance of CD8+ T cells, given the fact that most tumors express only MHC class I molecules and predominant effector cells are CD8+ CTLs. There is increasing evidence, however, that CD4+ T cells play a more direct role, beyond delivery of assistance in the generation of antitumor immunity [73]. In the priming phase, CD4+ T cells activate APCs through the interaction between CD40L and CD40, respectively, so that the educated APCs acquire the capacity to stimulate CD8+ T cells [74]. CD4+ T cells also function to maintain the numbers and cytotoxic capacity of CD8+ T cells and promote the infiltration of CD8+ T cells into tumors [75]. Through cross-priming, the fusion cells can activate antigen-specific CD4+ T cells that become multifunctional effectors producing IL-2, IFN-*γ*, IL-4, and IL-10 [14, 19, 64]. Moreover, the fusion cells also can function like APCs with the ability to migrate to draining lymph nodes, where they reside in the T cell area, interact with CD4+ and CD8+ T cells, and induce potent antitumor immunity [14, 27]. Both direct stimulation and cross-priming by host APCs participate in CD4+ and CD8+ T-cell activation by the fusion cells [14]. 

## 5. Antigen-Specific Polyclonal CTL Responses Induced by DCs/Tumor Fusions

The priming and expansion of polyclonal CTLs by vaccines has potential in vaccine applications for cancer. The goal of the vaccines is to prime the patient's own immune system to recognize and destroy the tumor without harming normal cells. Cancer vaccines that rely on induction of antitumor immunity against a single antigen are potentially subject to tumor-cell resistance mediated by downregulation of the single antigen. Therefore, antigen-specific polyclonal CTL responses have the potential to maximize the protection against various subsets of tumor cells with down regulation of certain tumor antigens, which may appear during the course of tumor progression. DCs/tumor fusions are potent inducers of antigen-specific polyclonal CD4+ T cells, which are essential for the induction of augmented polyclonal CTL responses against autologous tumor cells. Preclinical human studies have demonstrated that the fusions could induce antigens (CEA, MUC1, and WT1) specific CTLs simultaneously in HLA-A2- and/or -A24-restrictive elements in vitro [10, 19, 67, 76, 77]. Moreover, administration of the polyclonal CTLs could regress tumors in SCID mice and render mice free of disease up to the end of experiment [68]. In addition, DC/tumor fusion cells could be efficiently frozen without loss of either antigen presentation potency or T cell stimulatory capacity inducing polyclonal CTL responses [76]. The cryopreserved DC/tumor fusion cells have potential applicability in the field of antitumor immunotherapy and provide a platform for adoptive immunotherapy in the clinical setting.

## 6. Generation of Regulatory T Cells (Treg) by DCs/Tumor Fusions

Prevailing paradigms stipulate linear differentiation programs driving T cell lineage commitment, beginning with naive T cells that become Th1, Th2, Tregs, or Th17 depending on the cytokine milieu, where the T cells encounter at the time of antigenic stimulation. The presence of IL-12 causes naive T cells to differentiate into Th1 cells; IL-4 drives naive T cells to become Th2 cells; TGF-*β* drives them to become Tregs, and TGF-*β*, together with IL-6 and IL-21, promotes Th17 cell development [78, 79]. The cytokine milieu is associated with activated DCs, tumors, cancer-associated fibroblasts (CAF), or tumor associated macrophages (TAM). The control of immune-balance is essential for the cancer therapy. There is increasing evidence that DCs in situ induce antigen-specific unresponsiveness or tolerance in central lymphoid organs and in the periphery. The presentation of antigens to CD4+ or CD8+ T cells by immature or partially mature DCs results in tolerance [80] or induction of regulatory CD4+ and CD8+ T cells [81]. Tumors express or induce immunosuppressive cytokines such as TGF-*β* and IL-10. As a result, tumor-antigen cross-presentation by DCs induces T cell anergy or deletion and Treg instead of antitumor immunity [82]. Indeed, it has been reported that tumor progression correlated with an accumulation of immature DCs that induced the expansion of Tregs in lymphoid organs of tumor-bearing hosts [83]. Recently it has become possible to define Tregs on the basis of their expression of the transcription factor forkhead box protein 3 (Foxp3) [84]. Tregs have been shown to exert their effects through the activities of TGF-*β* [85], IL-10 [86], CTLA-4 [87], or through accumulation of IL-2 via expression of CD25 [88]. Tumor-derived TGF-*β* reduced the efficacy of DCs/tumor fusion vaccine via an in vivo mechanism [28]. The blockade of tumor-derived TGF-*β* reduced Tregs induction by the DCs/tumor fusions vaccine and enhanced antitumor immunity [38]. We have reported that the supernatant from human hepatocellular carcinoma (HCC) cells induced functional impairment of DCs as demonstrated by the downregulation of MHC class I and class II, CD80, CD86, and CD83 molecules [10]. Moreover, DCs exposed to the culture supernatants from HCC cells secreting TGF-*β* failed to undergo full maturation upon stimulation of TLR 4 agonist. Importantly, fusions of DCs and HCC cells generated in the presence of the culture supernatants from HCC cells promoted the generation of CD4+ CD25high Foxp3+ Treg and inhibited CTL induction. It has been demonstrated that CAF and TAM synthesized proteins, such as VEGF, TGF-*β*, and IL-10, all of which contributed to the local immunosuppressive environment [89, 90]. A major obstacle to the development of any active immunotherapeutic approach to cancer is the immunosuppressive environment by the growing tumor. Therefore, a combination of control of Treg and concomitant induction of efficient polyclonal CTLs may be a more effective immunotherapy to reduce recurrence and prolong survival.

## 7. Modified Fusions of DCs and Tumor Cells

While DCs/tumor fusions approach has been developed in animal studies, many adjuvants, including IL-2, IL-12, IL-18, and synthetic oligodeoxynucleotides (ODNs) containing specific bacterial unmethylated CpG motifs (CpG ODNs), have been used to enhance the ability of DC/tumor fusion vaccines to evoke antitumor immune responses [26, 29, 31, 54, 61]. These results suggest that the fusion vaccine needs to be modified to enhance antitumor immunity. The biggest advantage in DCs/tumor fusion strategy is that modifications of DCs as well as tumor cells are independently possible while their characters persist after the fusion. This is an important difference between the DCs/tumor fusion strategy and whole tumor lysates loading strategy. Therefore, the therapeutic efficacy of a vaccine requires the improved immunogenicity of both DCs and tumor cells. In the absence of proper costimulation, antigen presentation by DCs induces tolerance [1]. In particular, recent studies suggest that Toll-like receptor- (TLR-) agonist CpG ODNs or conserved pathogen-associated molecular patterns, such as penicillin-killed *Streptococcus pyogenes *(OK-432), start the DC maturation process, which is a critical event in the induction of full effector function in T cells. The DCs stimulated with the TLR agonist, OK-432 (OK-DCs), show higher expression levels of MHC class I and class II, CD80, CD86, CD83, IL-12, and heat shock proteins (HSPs) than do immature DCs [76]. On the other hand, the immunogenicity of tumor cells can be improved by heat-treatment [91]. Heat-treated autologous tumor cells display a characteristic phenotype with increased expression of HSPs, carcinoembryonic antigen (CEA), MUC1, and MHC class I [77]. Intracellular HSPs play an important role as molecular chaperones in cellular protein-folding pathways [92]. Moreover, cross-priming is based on the transfer of proteasome substrates that are transcriptionally upregulated by heat treatment in human tumor cells [91, 93]. In contrast, extracellular HSPs act as chaperon peptides and interact with DCs in a receptor-mediated manner, leading to maturation as well as proinflammatory responses [91, 94], all of which are likely to be key danger signals to the antitumor immune system. Therefore, we have created fusions of OK-DCs and heat-treated tumor cells to elicit potent antitumor responses. The modified fusions show to be active as demonstrated by (1) up-regulation of multiple HSPs, MHC class I and class II, CEA, CD80, CD86, CD83, and IL-12; (2) activation of CD4+ and CD8+ T cells able to produce IFN-*γ* at higher levels; (3) efficient induction of antigen-specific polyclonal CTL activity against tumor targets; and (4) superior abilities to induce CD107+ IFN-*γ* + CD8+ T cells and CD154+ IFN-*γ* + CD4+ T cells. These fusions may provide a promising means of inducing therapeutic antitumor immunity.

## 8. From Autologous to Allogeneic Tumor Cells for DCs/Tumor Fusions

**Figure 2:**
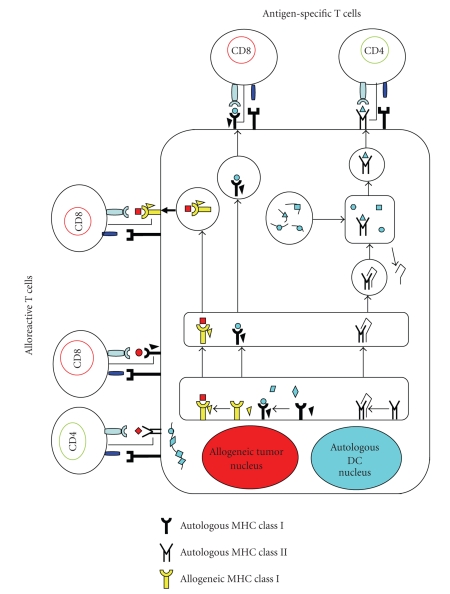
Fusions of autologous DCs and allogeneic tumor cells (DCs/allo-tumor). The DCs/allo-tumor can stimulate both CD4+ and CD8+ T cells as same as fusions of autologous DCs and autologous DCs (DCs/auto-tumor). Moreover, the DCs/allo-tumor can also stimulate alloreactive T cells due to the presence of allogeneic HLA class I molecules from allogeneic tumor cells. Autologous MHC molecules present foreign peptide derived from allogeneic tumor cells to T cell selected to recognize self MHC-foreign peptide complexes. In addition, T cell also can recognize an allogeneic MHC molecule whose structure resembles the self MHC-foreign peptide complexes and structure formed by both the allogeneic MHC molecules and the bound peptide.

As a fusion partner, the advantage of using autologous tumor cells is their possession of all the relevant TAAs required for mounting effective antitumor immunity. However, in the clinical setting of the patients with cancer, a major difficulty for the DCs/tumor fusion vaccine is the preparation of sufficient amounts of autologous tumor cells because of both the availability of limited tumor samples and the difficulty in culturing tumor cells. It has been reported that hybrid cells generated by fusing DC from healthy donor with allogeneic tumor cell line have induced CTL responses against the allogeneic tumor cells used for fusion [95, 96]. The basis for using allogeneic tumor cell lines instead of autologous tumor cells is that some antigens are shared by most of tumors. We have reported that fusions generated by autologous DCs and allogeneic tumor cell lines can induce antigen-specific polyclonal CTLs with cytotoxic activity against autologous tumor cells (Figure 2) [67, 68]. This strategy has numerous advantages. (a) Allogeneic tumor cell lines are well characterized as TAA source. (b) Allogeneic tumor cell lines, which shared with TAAs, can grow well in vitro; thus, there is no limiting factor for preparation of tumor cells. (c) It is not necessary to determine HLA typing of patients and allogeneic tumor cells as a partner of fusion cells, because autologous dendritic cells can process and present multiple TAAs from allogeneic tumor cells in the context of MHC class I and class II. Indeed, allogeneic tumor cells (melanoma and prostate cancer), transduced with granulocyte-macrophage colony-stimulating factor (GM-CSF), have been applied clinically and shown to induce antitumor immunity [97, 98]. In this trial, whole allogeneic tumor cells were genetically modified to secrete the immune stimulatory cytokine, GM-CSF, and then irradiated to prevent further cell division. After phase III trials evaluating an allogeneic GVAX immunotherapy in prostate cancer were finished, the trials have been suspended. While currently explored allogeneic approaches in whole tumor cell-based vaccination procedures represent an improvement in terms of standardization over their autologous counterparts, they nevertheless entail the culture of large batches of cells under good manufacturing practice (GMP) grade conditions [99]. Further optimization of these in vitro culture methodologies is required. A major challenge to develop an allogeneic tumor cell-based vaccine strategy is to overcome the potential hazards of fetal calf serum (FCS) that limit safety in clinical trials.

## 9. From Autologous to Allogeneic DCs for DCs/Tumor Fusions

The rationale for using allogeneic DCs as a fusion partner is based on the finding that a high frequency of unprimed T cells from an individual react against the foreign MHC antigens of another individual. Additional potential benefit of using allogeneic DCs is that DCs from healthy donors are readily available in unlimited amounts. DCs from cancer patients may be defective in APC function, owing to cancer treatment, such as chemotherapy and irradiation. It has been demonstrated that fusions of both autologous and allogeneic DCs are effective in inducing antitumor immunity in human and animal models [100, 101]. The allogeneic DCs/autologous tumor fusions express DCs-derived allogeneic HLA class II molecules and HLA class I molecules derived from both DCs and tumor cells [102–104]. There are mainly four cases using allogeneic DCs for fusions-based vaccine. (a) Where there is no sharing of any MHC molecules between allogeneic DCs and autologous tumor cells, autologous MHC-class I restricted presentation of tumor peptides by the DCs through cross-presentation is not possible. (b) Where there is sharing of MHC class I molecules, autologous MHC-class I restricted presentation of tumor peptides by the DCs through cross-presentation is possible. The direct CD4+ T cell response to the allogeneic MHC class II antigens on allogeneic DCs will provide potent T cell help for the generation of antigen-specific CD8+ CTL responses to autologous tumor peptides presented by the shared MHC class I molecules. (c) Where there is sharing of MHC class II molecules, autologous MHC-class IL restricted presentation of tumor peptides by the DCs through cross-presentation is possible. The direct CD4+ T cell response to the semi-allogeneic MHC class II antigens on semi-allogeneic DCs will provide potent T cell help for the generation of antigen-specific CD4+ T cell response to autologous tumor peptides presented by the shared MHC class II molecules. (d) Where there is some sharing of both MHC class I and class II molecules, the fusions express allogeneic MHC class I and class II molecules derived from semi-allogeneic DCs for direct stimulation of the patient's CD4+ T cells as well as all the patient's HLA class I and class II molecules for autologous MHC-restricted tumor peptide presentation. The alloreactive T cell response by semiallogeneic fusions might help for the initiation and expansion of antigen-specific responses to autologous tumors by autologous MHC-restricted elements. Therefore, where there is no sharing of MHC molecules between allogeneic DCs and autologous tumor cells, efficient antitumor immunity may not be induced in therapeutic experiment [51]. Semiallogeneic fusions may be effective to induce antigen-specific polyclonal CTL responses. Indeed, semiallogeneic fusions elicited a significantly stronger antitumor immunity than did by syngeneic fusions in animal studies [31, 34]. However, it has also been reported that expression of self MHC by semiallogeneic fusions could induce antigen-specific immunity; however, concurrently activated allogeneic bystander responses do not provide helper or adjuvant effects [105]. In clinical trials, both autologous and allogeneic DCs fused with autologous tumor cells have been more effective as vaccines in the induction of CTL responses and antitumor activities [106, 107]. 

## 10. HSP70-Peptide Complexes Derived from DCs/Tumor Fusions

Heat shock proteins (HSPs) play a primary role as intracellular molecular chaperones in the pathways of antigenic protein folding within the cell [108]. The HSP/peptide complexes can be taken by DCs through receptors and presented in MHC class I and class II molecules on DCs [109]. This phenomenon leads to activation of maturation and representation of peptide antigen cargo of HSPs by DCs and initiates antigen-specific polyclonal CTL responses [92]. In several clinical trials, autologous HSP/peptide complexes have induced CTLs against autologous targets [110]. To improve the potency of chaperone protein-based vaccine, we have produced an improved HSP70-based vaccine with the use of DCs/tumor fusions [111]. The HSP70/peptide complexes (HSP70.PC) derived from DCs/tumor fusions were especially different from those derived from tumor cells in enhanced association with immunologic peptides in animal models. The HSP70.PC derived from the fusions have increased their immunogenicity and therefore may constitute an improved formulation of chaperone protein-based tumor vaccine. Recently, it has been also reported that human DCs pulsed with HSP70.PC extracted from DCs/tumor fusions enhanced CTL responses significantly more than that obtained from DCs pulsed with HSP70.PC from DCs pulsed with tumor cell lysates [112]. Therefore, this is an alternative molecular chaperone-based cancer vaccine using DCs/tumor fusions. Future studies should be required to improve the field of the chaperone-based cancer vaccine.

## 11. Clinical Trials

Based on the unique features of fusion cell based vaccines and the observations of tumor eradication in animal studies, initial Phase I/II clinical trials with fusion vaccines have been conducted in a variety of tumors (Table 1). Fusions vaccination was first reported in patients with melanoma [113, 114]. The fusions of allogeneic DCs and autologous tumor were irradiated and injected subcutaneously as a vaccine. Seven of the 16 patients responded to the vaccination, one with complete response, one with partial response, and five with stable disease following to previous rapid progression. Similar results in patients with melanoma were reported from another group [116, 117]. In our initial clinical trials of fusions-based vaccination, eight patients with malignant glioma were treated with fusions of autologous DCs and autologous tumor cells. Vaccination with fusions resulted in immunological responses and two patients showed partial responses, indicating that limited success has occurred in clinical trials. To enhance clinical responses, we had conducted a Phase I/II clinical trials for the vaccination with low dose of recombinant human (rh) IL-12 in patients with malignant brain tumor, gastric, colorectal, ovarian carcinoma, and melanoma [115, 118]. Eleven out of 15 patients with malignant glioma achieved a stable response and 24 patients had a progressive disease after 8 weeks of the initial treatment [115]. No serious adverse effects were observed. In four patients, magnetic resonance imaging showed a greater than 50% reduction in tumor size. One patient had a mixed response. Therefore, administration of fusions and rhIL-12 can induce more effective antitumor effects than fusions alone in some patients with malignant glioma. These data are compatible with the results from mouse brain tumor model in which administration of fusions and rIL-12 markedly prolonged the survival of mice with brain tumors compared with fusions or rIL-12 alone [119]. Moreover, vaccination of autologous fusions alone in patients with breast, renal, colorectal, and gastric cancer resulted in immunological responses. Interestingly, two out of 10 patients with metastatic breast cancer exhibited disease regression, including a near complete response of a large chest wall mass [17]. Five out of 13 patients with renal carcinoma and one out of 10 patient with breast cancer had disease stabilization [17]. This group has also evaluated the effect of vaccination with fusions of allogeneic DCs and autologous tumor cells in patients with renal cell carcinoma [106]. Vaccination of patients with stage IV renal cell carcinoma with allogeneic DCs/autologous tumor fusions resulted in immunologic and clinical responses in a subset of patients. Two out of 21 patients demonstrated a partial clinical response and 8 patients with had stabilization of their disease. In clinical trials, only limited therapeutic results are obtained. One of the reasons is that all patients were in advanced stage and extremely small amounts of fusions are used. Fusions-based vaccine may work more effectively in patients in the early stage of the disease with low tumor burden. Moreover, patients with a still uncompromised immune system are expected to respond best to the vaccine. Fusions-based vaccine may be used in combination with conventional therapies, including surgery, chemotherapy, or irradiation. 

## 12. Future View

The DCs/tumor fusions vaccine has been successfully used in mice models. Moreover, human DCs/tumor fusions have enormous potential activities to induce polyclonal CTL responses against autologous targets in vitro. However, the overall rate of clinical responses remains to be low. Fusions vaccine alone may be insufficient to have a significant contribution to treat advanced cancer patients. There are increasing evidences that tumor-derived soluble factors promote the induction of tolerance through the generation of CD4+ CD25highFoxp3+ Treg subset, which is linked to compromised immune responses in patients with advanced cancer [120]. Moreover, it has recently been reported that DCs are capable of inducing conversion of naive CD4+ T cells to adaptive CD4+ CD25+ Foxp3+ Treg in the presence of TGF-*β* or IL-10 derived from tumor cells [121]. We have also reported that soluble factors derived from tumor cells promoted the generation of CD4+ CD25high Foxp3+ Treg and inhibited CTL induction by fusions [10]. The elimination of immunosuppressive immune cells, including Tregs, myeloid derived suppressor cells (MDSCs), or tumor-associated macrophages (TAMs), may improve clinical responses. In animal study, enhancement of antitumor immunity can be induced with a combination of fusions and regulatory T cell depletion in pancreatic cancer bearing mice [41]. More importantly, in a Phase I/II clinical trial, partial removal of Tregs can further enhance DC vaccine-induced immune responses in cancer patients [122]. The combination of direct enhancement of CTL function and concomitant inhibition of Treg function through blockade of cytotoxic T lymphocyte-associated antigen 4 (CTLA-4) on both cell types is essential for mediating the full therapeutic effects of anti-CTLA-4 antibodies in cancer immunotherapy. Another approach would be to enhance T cell costimulation by administering agonistic antibodies specific for 4-1BB [123], OX40 [124], cytotoxic T lymphocyte-associated antigen 4 (CTLA4) [125], or programmed death 1 (PD-1) [126]. It has been reported that a common pathway of endogenous OX40 interaction is critical for the development of a therapeutic immune response by fusions vaccination [127]. The pathological interactions between cancer cells and host immune cells in the tumor microenvironment create an immunosuppressive network that promotes tumor growth, protects the tumor from immune attack, and attenuates immunotherapeutic efficacy. Therefore, it is also essential to develop interventions that counter the propensity of tumors to evade immune elimination, such as immunization against the tumor stroma cells [128].

## Figures and Tables

**Table 1:** Asessment of the fusions vaccine.

	Fusions					
Tumor	Dendritic cell	Tumor	Adjuvant	Patients (n)	Clinical response	Ref.

Melanoma	Allogeneic	Autologous		16	1 (CR)	[113, 114]
					1 (PR)	
					5 (SD)	
					9 (PD)	

Glioma	Autologous	Autologous		8	2 (PR)	[115]
					1 (SD)	
					5 (PD)	

Melanoma	Autologous	Autologous		17	1 (PR)	[116]
					1 (SD)	
					15 (PD)	

Melanoma	Allogeneic	Autologous	rh IL-2	11	1 (SD)	[117]
					10 (PD)	

Glioma	Autologous	Autologous	rh IL-12	12	3 (PR)	[115, 118]
					2 (MR)	
					4 (SD)	
					3 (PD)	

Breast cancer	Autologous	Autologous	rh IL-12	2	1 (SD)	[115, 118]
					1 (PD)	

Gastric/Colorectal carcinoma	Autologous	Autologous	rh IL-12	3	1 (SD)	[115, 118]
					2 (PD)	

Ovarian carcinoma	Autologous	Autologous	rh IL-12	3	2 (SD)	[115, 118]
					1 (PD)	

Melanoma	Autologous	Autologous	rh IL-12	4	4 (PD)	[115, 118]

Breast cancer	Autologous	Autologous		10	2 (PR)	[17]
					1 (SD)	
					7 (PD)	

Renal cell carcinoma	Autologous	Autologous		13	5 (SD)	[17]
					8 (PD)	

Renal cell carcinoma	Allogeneic	Autologous		20	2 (PR)	[106]
					8 (SD)	
					10 (PD)	

Hepatocellular carcinoma	Autologous	Autologous		1	1 (PD)	[10]

Renal cell carcinoma	Allogeneic	Autologous		10	1 (PR)	[107]
					6 (SD)	
					3 (PD)	

CR: complete response; PR: partial response; MR: mixed response; SD: stable disease; PD: progressive disease.
